# Third-Generation Platelet Concentrates in Periodontal Regeneration: Gaining Ground in the Field of Regeneration

**DOI:** 10.7759/cureus.28072

**Published:** 2022-08-16

**Authors:** Unnati Shirbhate, Pavan Bajaj

**Affiliations:** 1 Periodontics and Implantology, Sharad Pawar Dental College, Datta Meghe Institute of Medical Sciences, Wardha, IND

**Keywords:** prf, platelet concentrate, growth factors, l-prf, plasma, centrifugation, rpm, i-prf, t-prf, a-prf

## Abstract

Platelets are important for hemostasis and the healing of wounds. In clinical settings, healing cytokines including insulin-like growth factors (IGF), platelet-derived growth factors (PDGF), and transforming growth factors (TGF) are commonly implemented. The regenerative approach in dentistry frequently employs platelet concentrates (PCs) that are “autologous in origin” and have a high concentration of platelets, growth factors, and leukocytes. First-generation PCs is made of platelet-rich plasma (PRP), while second-generation PC is made of platelet-rich fibrin (PRF). Both have limitations, so modification protocols and development in PRP and PRF derivatives are required for advancement mechanisms, strength, biodegradability, retention ability in the field of regenerative dentistry, and so on. As third-generation PC, newer genera kinds of PRF, such as advanced-PRF (A-PRF), advanced-PRF+ (A-PRF+), injectable-PRF (i-PRF), and titanium-PRF (T-PRF), were introduced. A-PRF matrices in their solid form were introduced using the low-speed centrifugation concept (LSCC). The applied relative centrifugal force (RCF) for A-PRF is reduced to 208 g as a result of this improved preparation process. A-PRF features a greater number of neutrophil granules in the distal region, especially at the red blood cells-buffer coat (RBC-BC) interface, and the A-PRF clot has a more porosity-like structure with a bigger interfibrous space than PRF. Since the PRF is in a gel form and is difficult to inject, i-PRF was formulated to address this problem. Compared to the other two protocols, the i-PRF protocol requires far less time, and this is the advantage of this PC. This is because i-PRF just needs the blood components to be separated, which happens within the first two to four minutes. Compared to normal L-PRF, T-PRF creates fibrin that is thicker and more densely woven. Titanium has a higher hemocompatibility than glass, which could lead to greater polymerized fibrin formation. In periodontal regenerative operations, oral surgery, and implant dentistry, PRF and its newer advanced modifications have demonstrated promising results and desirable results in both soft and hard tissue regenerative techniques.

## Introduction and background

Platelets are important for hemostasis and healing of wounds. Platelet-derived growth factor (PDGF), transforming growth factor (TGF), and insulin-like growth factor (IGF) are proteins that are stored intracellularly and are vital for wound healing [1]. Healing cytokines can be obtained from platelet growth factors, which are a common source in clinical conditions [1]. Bone remodeling, wound healing, hair regrowth, nerve regeneration, aging facial skin, acne scarring, and diabetic wounds are just a few of the regenerative medicine applications for these bioactive chemicals [2].

Platelet concentrates (PCs) have become popular in dentistry as a regenerative therapy since they are autologous in origin, and they deliver a high concentration of platelets, growth factors, and leukocytes (i.e., 90% of the platelets and 50% of the leukocytes when compared to the concentration from natural blood). PCs were created and developed to make “human blood proteins as a source of growth factors capable of inducing angiogenesis and tissue ingrowth,” which is based on the notion that tissue will regenerate and will need a blood supply to regenerate [3]. Marx's initial clinical report in dentistry was the start of the first generation, with the primary purpose of validating the concentration of platelets and growth factors in PRP preparations. Anticoagulants found in blood by-products, such as acid citrate dextrose-A (ACD-A), are a critical component of first-generation PC research [4].

## Review

First- and second-generation platelet concentrates

In second-generation research, Choukroun generated a slew of platelet-rich plasma (PRP) derivatives, including platelet-rich fibrin (PRF) [4]. PRP was created by coagulating the whole form of blood samples with the presence of anticoagulants in it, then adding coagulation factors as needed. These intricate processes demand skillful operators because the presence of anticoagulants or coagulator agents may have negative effects on the tissue which is to be regenerated (see Table 1 for content by PC generation) [4]. In the case of PRF, 10 ml of blood sample should be collected in glass coating tubes and centrifuged for 12 minutes at (around 400 gm) 2,700 rpm, and it can be made without using anticoagulants or coagulation agents by the stimulatory effect of the intrinsic coagulation cascade or intrinsic pathway. This procedure also allowed clinicians to save time and effort when it came to PC preparation. This generation can entail the development of freeze-dried PRP [4].

**Table 1:** Fundamental platelet concentrate (PC) history and study Source: Reference [4].

Generations (PC)	Major platelet concentrate contents
First generation	The concentrated count of platelets and growth factors has been validated. Devices for automated PRP preparation are being developed.
Second generation	Modifications to the prior preparatory techniques and the development of new platelet-rich plasma derivatives, like platelet-rich fibrin, are being pursued (i.e., PRF).
Third generation	Platelet-rich plasma derivatives are compared in terms of their ability to react, retain, and release various growth factors for their mechanical stability, strength, and biodegradability.
Fourth generation	PCs' coupling (partner cells) is being investigated.

Third-generation platelet concentrates

PRF obtained in a gel formulation makes it challenging for injection. As a result, advancements in the field of PRF have been made to overcome these restrictions, and fresh kinds of PRF have been introduced as third-generation PC (see Table 2 for advanced-PRF, advanced-PRF+, injectable-PRF, and titanium-PRF) [5].

**Table 2:** Advancement and evolution in third-generation platelet concentrates and procedure Source: Reference [5].

Platelet concentrate (Year)	Procedure for centrifugation
Advanced: Platelet-rich fibrin or A-PRF (2014)	In sterilized simple glass-based vacuum tubes, spin at 1500 rpm for 14 minutes.
Advanced: Platelet-rich fibrin plus or A-PRF+ (2016)	In sterilized simple glass-occupied vacuum tubes, spin for 8 minutes at 1300 rpm.
Injectable: Platelet-rich fibrin or I-PRF (2015)	In plastic tubes, spin for 3 minutes at 700 rpm.
Titanium: Platelet-rich fibrin or T-PRF (2014)	In medical-grade titanium test tubes, spin for 12 minutes at 2800 rpm.

Advanced platelet-rich fibrin

Background

Platelet-rich fibrin (PRF) is generated by Choukroun from the blood that has not been processed with anticoagulants. Ghanaati et al. used histological cell detection with the histomorphometric cell distribution measurements for the standard platelet-rich fibrin (S-PRF; centrifuged for 2700 rpm, 12 minutes) and advanced platelet-rich fibrin (A-PRF; centrifuged for 1500 rpm, 14 minutes) protocols to compare their effects of centrifugation force (speed and time) on the distribution of blood cells, which is important for wound healing and tissue regeneration. When the rpm was decreased while the centrifugation time was prolonged in the A-PRF group, the number of neutrophil granulocytes in the distal region of the clot was observed to be increased in advanced-PRF. Neutrophils were primarily located close to the interface between both the red blood cells (RBCs) and buffer coat (BC) in the S-PRF group. Monocytes are assisted in maturing into macrophages by the neutrophilic granulocytes [6]. From this result, a larger quantity of these definite cells could affect how host macrophages, as well as monocytes within its clot, differentiate after the implantation. It results as above that, when the monocytes and the macrophages and their growth factors used to be present, A-PRF may have a definite effect on the bone and the soft tissue regenerations [6].

Preparation of A-PRF

A venous sample of 10 mL of blood was collected from the person's hand, i.e., antecubital vein, and transferred to glass tubes right away. To begin platelet activation and fibrin polymerization, there was no anticoagulant in the tubes. The tubes of the A-PRF group underwent 14-minute centrifugation at 1500 rpm. The final tube product was composed of the following three layers: the very first layer that is of acellular plasma, A-PRF clot that lies in the center, and RBCs that last at the bottom [7].

Growth Factors

Transforming growth factor (TGF-1), vascular endothelial growth factor (VEGF), PDGF, epidermal growth factor (EGF), and insulin-like growth factor (IGF1) have significantly larger total amounts of secreted growth factors in A-PRF [6].

Clinical Applications

When compared to PRF, A-PRF improves periodontal weakened sites by regenerating tissues by increasing parameters like probing depth, lowering relative attachment loss, and increasing bone height, which is similar to infrabony pocket treatment [8]. A-PRF improves soft tissue recovery at extraction sites and reduces postoperative discomfort and the requirement for analgesics [7]. The increase of VEGF expression by A-PRF had a beneficial effect on the angiogenesis of the gingiva. As a result, A-PRF may be advantageous in the regeneration of gingival tissue [9]. A-PRF can assist with the healing of a wound, tissue repair, and regeneration. A-PRF appears to be a good and perfect source of autologous cells that can stimulate each other, resulting in a synergistic interaction for tissue regeneration [6]. A-PRF serves as a scaffold material and a better reservoir for delivering specific growth elements to the application site [10]. A-PRF can be used to aid in palatal wound healing following the harvesting of free gingival grafts [11]. Guided-tissue regeneration (GTR) surgical approach for the severe involvement of bone defects was assisted using a three-dimensional printing model and A-PRF technology [12].

Advanced platelet-rich fibrin plus

Background: A new preparation or formulation known as advanced-rich plasma plus (A-PRF+) was introduced as a result of additional modifications to the A-PRF technology. Researchers attempted to reduce the centrifugal duration; hence, the overall amount of force can result in cell loss as the centrifugal force directly influences the number of cells trapped inside the PRF matrix [13].

Preparation of A-PRF+

As per Pavlovic et al.'s study, the formulation of the A-PRF+ preparation routine was established by Fujioka-Kobayashi et al. by lowering the centrifugation speed to 1,300 rpm at 200 g with a centrifugation time of eight minutes [13].

Growth Factors

The A-PRF+ has a substantially greater level of released growth factors in it than the A-PRF and leukocyte-PRF, which are TGF-1, VEGF, PDGF, EGF, and IGF1. Human gingival cells migrated and proliferated more as a result of A-PRF+ [13].

Clinical Applications

As a human autologous product, A-PRF+ has the potential to improve periodontal repair and is effective in treating intrabony periodontal defects [14]. Membranes prepared from the A-PRF+ with significantly increased maximal traction indicate a higher strength and viscoelasticity in periodontal and oral surgeries [15]. A-PRF+ is having a higher potential and is an adjunct to the standard approach for regenerative treatment in the treatment of socket preservation [16]. In the management of alveolar osteitis, A-PRF+ represents a more effective and faster regeneration and helps in the development of hard and soft tissue healing as well as pain alleviation [17]. In endodontic surgeries, A-PRF+ use is a cost-effective and safe way to improve postoperative quality [18].

Injectable platelet-rich fibrin

Background

The new formulation creation of an injectable-PRF (known as i-PRF) aims to provide clinicians with a liquid formulation of easily usable platelet concentration that can be used to be employed alone as a regenerative agent or in addition to a variety of biomaterials for regeneration. In i-PRF, regenerative cells with a greater number of concentrations of the growth factors are more prevalent and might be seen because of the slower and shorter centrifugation speed [19].

Preparation of i-PRF

According to the preparation of Mourao et al., 9 mL of autologous blood was collected without taking any extra preservatives in a test tube and then centrifuged at 3300 rpm force for two minutes, yielding an orange-colored fluid in the i-PRF tube [19,20]. As stated by Miron et al., autologous blood was collected without any anticoagulants or preservatives into plastic tubes and centrifuged at a force of 700 rpm for three minutes. Plastic-coated tubes are hydrophobic in nature and have a hydrophobic surface that prevents the coagulation process from working properly. As a result, due to the centrifugation force, all of the clotting factors and platelets essential for the formation of PC will reach the upper zone of a tube within the very first two to four minutes. The upper layer contains separated plasma and platelets, which are pale yellow or orange and are used in an injectable form [19]. According to Al-Maawi et al., blood was collected in the tube and centrifuged at 600 rpm for eight minutes [20]. After centrifugation, i-PRF was generated, with yellow orange-colored blood [19]. A centrifuge machine was used at 2,700 rpm for three minutes to collect blood-filled test tubes according to Castro et al. and Cortellini et al. [19]. Miron et al. employed a horizontal centrifugation procedure at 200 g for eight minutes to prepare i-PRF [19].

Growth Factors

It has been found that i-PRF can promote an increased rate of migration of fibroblast and the expression of the growth factors of PDGF, TGF, and collagen1 as well as release higher quantities of several growth factors [20].

Clinical Applications

i-PRF is a potential regenerative supplement to dental operations [21]. i-PRF is a good material for improving the handling qualities of bone grafts in periodontal defects, atraumatic extraction operations, and endo-perio lesions [22]. Local administration of i-PRF along with scaling and root planing (SRP) improves periodontal health while treating chronic periodontitis as opposed to SRP alone [23]. The i-PRF is used in knee replacement, facelifts, reducing the risk of infections after heart surgery, sports-related injuries, tendon or ligament injuries, cases of osteoarthritis, meniscus repair, treatment of alopecia, regenerations in the musculoskeletal operations, and treatment of acne [24]. In patients who are having thin gingival phenotypes, gingival recessions, infrabony defects, furcation defects, bone regeneration, local drug delivery, and root coverage, the applications of the i-PRF may increase the success rate of the periodontal regenerative therapy [20]. This improves the bone quality and can be utilized to grafting dental implants [21].

Titanium platelet-rich fibrin

Background

Titanium has strong hemocompatibility and is non-corrosive. These are important characteristics of the biomaterials that interact with the blood. Both titanium and glass tubes stimulated platelets identically, and the clot formed in the titanium tubes was seen clinically to be equivalent to the clot that is formed in glass tubes. Titanium-PRF's fibrin structure appeared to be more closely woven and thicker. The titanium-formed fibrin carpet has a firmer network structure. It is critical to have a strong fibrin structure to delay the time it takes for fibrin to resorb and increase the time it takes for growth factors to be released [25]. T-PRF is also used to prevent problems with silica contamination as well as prevent any short-term and long-term detrimental effects from dry glass-containing tubes or plastic tubes that are glass coated [25].

Preparation of T-PRF

Antecubital veins from the patient's arm are used to withdraw a 10 mL blood sample, and the collected blood was then transferred to a grade IV of titanium tube. Then the titanium tubes were centrifuged immediately at a centrifugal force of 3000 rpm for 10 minutes at room temperature at a specified table [26]. The T-PRF clot was retrieved from the tubes using sterile tweezers and separated from the base of the RBC with sterile scissors and then centrifuged before being placed on sterile woven gauze. On sterile woven gauze, the clots are kept for 20 minutes so that their serum will slowly release [26].

Growth Factors

It is observed that T-PRF can release a greater number of concentrations of the growth factors such as VEGF, PDGF, TGF, EGF, IGF-I, and hepatic growth factor (HGF) [26].

Clinical Applications

Periodontal procedures such as osseous defects and furcation diseases are discussed in a study by Mitra et al. [27]. The use of T-PRF in sinus lift treatments was evaluated in the study by Olgun et al. [28]. T-PRF can be utilized as autogenous material as a substitute for connective-tissue graft (CTG), a recognized gold standard for root coverage [29]. T-PRF improved clinical metrics, suggesting that they may help in the restoration of soft tissue in intrabony lesions [30]. When combined with open flap debridement (OFD), the use of T-PRF membranes leads to a significant release of the greater number of growth factor concentrations and a lower number of ‘RANKL/OPG ratio’ in GCF [31]. T-PRF helps gain attachment level and lower pocket depth while treating endo-perio lesions [32].

Newer advances

In the future, procedures like leukocyte-rich PRF, which are economical, simple, and efficient, will be widely used in implant dentistry. PRP in gel formulation and PRF is an innovation with numerous potential clinically in periodontal surgeries and dentoalveolar surgeries that need to be researched and tested in regenerative dentistry.

PRF Lysate (PRF-Ly)

A relatively recent PRF product is the use of PRF lysate. The PRF lysate, which is the exudate, is collected after the PRF preparation and is incubated in a humidified atmosphere at 37°C. It is composed of 5% of CO2 and 95% of air. Growth factors like PDGF, TGF, VEGF, and EGF are all regarded to be beneficial. It has also been used to significantly increase proliferation rate, migratory rate, and collagen deposition rates to levels comparable to normal fibroblasts to repair the damages caused by chronic exposure to the UV rays to human cutaneous fibroblasts. More research is required to evaluate the utility of this novel use [33].

Lyophilized-PRF (Ly-PRF)

Fabrication: According to Ngah et al., the Ly-PRF fabrication method is proposed and based on the methods of Li et al. (2014) and Kardos et al. (2018) [33]. This technique was chosen primarily because Ly-PRF has been successfully used as a biomaterial for cranial bone repair in vitro and in vivo. This is the first time that a method for fabricating Ly-PRF has been documented in the literature, and comparable techniques were used in this investigation to ensure consistency. The Ly-PRF developed in this work had a sponge-like look in terms of physical features. Ly-PRF is a simple, cost-effective, and organic method for producing a PC with continuous growth factor release for bone regeneration applications. The Ly-PRF showed adaptability as a viable biomaterial for application as a craniofacial bioscaffold. Ly-PRF demonstrated fundamental scaffold qualities in addition to its particular properties as a reservoir for the growth factor PDGF-AB [33].

Albumin PRF (Alb-PRF)

It is a blood by-product produced in two processes after centrifugation; this includes heating and its incorporation (heating of the serum, low platelet plasma, and incorporation of cells). It is formed wholly from autologous blood (where the growth factor/GF and PRF cytokines are liquid, removed from the junction of the leukocyte zone and the RBCs). Translational research on this newest biomaterial is already progressive nowadays and has already undergone in vitro testing. Alb-PRF is anticipated to offer great results in facial medicine, aesthetic surgery, and oral/periodontal surgery [34].

The discovery of PCs that hang on to the growth factors intertwined in the fibrin network and release them over time expedites the wound-healing process. It is one of the most exciting recent newer advances in the field of regenerative approaches in dentistry. Platelets activate the coagulation cascade and create a stable clot after an injury, releasing growth factors that promote healing and tissue creation. Numerous autologous platelets concentrate techniques have been created and employed in the field of periodontal regenerative surgery. PRP was developed as the ideal growth factor delivery technique at the site of injury because it combines the fibrin-sealing properties of platelets with the effects of their growth factors. Because the growth factors are released for a very short period and different platelet concentrations have varying storage durations, the PRP preparation technique's lack of homogeneity is a limitation. Future research on PC for topical application in dental surgery has expanded significantly in recent years, notably in periodontology and implant dentistry. Injectable PRPs are likely to become more widely used in many other disciplines of surgery in the future, particularly in aesthetic dentistry and sports medicine [35].

## Conclusions

For a long time, platelet concentrations have been used in dentistry for a variety of applications. Due to the developments in this field's technology, the utilization of PRF in the periodontal regeneration approaches has improved. There has been numerous research done to evaluate the effectiveness of PRF in various treatments such as in periodontics, oral surgeries, and dental implants, with encouraging and promising results in both soft and hard tissue regeneration. The excellence and quality of fibrin scaffold are influenced by a variety of elements such as rotation speed, centrifugal unit duration, temperature, and blood hematocrit values. As a result, the important and exact role of leukocytes or fibrin in PRF scaffolds is regarded as a never-ending potential avenue for the near future periodontal regeneration research.
